# THE HOSPITAL TO HOME TRANSITION: EFFECTS OF LIFE CHANCES AND LIFE CHOICES ON UNPLANNED HOSPITAL READMISSIONS

**DOI:** 10.1093/geroni/igad104.2064

**Published:** 2023-12-21

**Authors:** Dale Yeatts, Cynthia Cready

**Affiliations:** University of North Texas, Denton, Texas, United States; University of North Texas, Denton, Texas, United States

## Abstract

Roughly 18% of all patients discharged from hospitals in the United States experience an unplanned hospital readmission (UR) within 30 days which can be life-threatening to patients and extremely costly for the health care system. Medicare alone paid $900.8 billion in 2021, or 21 percent of total national hospital expenditures. A promising theory for adding to our understanding of the causes for URs is Health Lifestyle Theory (HLT). It proposes that structural factors (e.g., SES, hospital procedures) affect one’s life chances and that socialization and personal experiences affect one’s life choices and the two merge to produce a person’s health lifestyle. The purpose of our study was to consider the ability of HLT to explain why some patients recover after hospital discharge while others experience URs. To accomplish our purpose, we interviewed 37 patients in their homes who had been discharged from a regional hospital in north Texas roughly 30 days prior to being interviewed. All the patients had been admitted for various serious illnesses. Fourteen of the patients had experienced an UR. Qualitative analyses included open and focused coding. Data from those experiencing an UR and those avoiding an UR were analyzed separately. We found the HLT to provide sound reasons for patients experiencing recovery or URs. Those who experienced an UR were found to have similar life chances caused by structural factors and similar life choices based on their socialization and personal experiences. Specific structural factors and personal experiences associated with life chances and life choices are presented.

